# Artificial Prions Created from Portable Control Elements

**DOI:** 10.1371/journal.pbio.0020093

**Published:** 2004-03-23

For decades, scientists accepted that the nucleic acids, DNA and RNA, packed with thousands of protein-coding genes, were the sole purveyors of genetic information; all inherited traits, from eye color to shoe size, must be stored and expressed through nucleic acid mechanisms. But prions are an exception. These misshapen proteins are capable of growing, replicating, and infecting other cells—that is, they are heritable. And all without a scrap of DNA. Most famous as the culprit behind bovine spongiform encephalopathy, or mad cow disease, prions also occur naturally in some organisms and may play important roles in their growth and development.

Prion-forming proteins normally exist as benign cellular components, such as enzymes or receptors. But they possess the innate ability to alter their three-dimensional structure, or fold, which changes their function and makes them almost impossible to destroy. Like other misfolded proteins, such as those responsible for Alzheimer's and Huntington's diseases, prions pack together and form aggregates. But what distinguishes prions from simple protein aggregates is their exponential growth and amplification, which allows them to infect new host cells. Prions grow by inducing normal proteins to alter their shape and adhere to an initial aggregate “seed.” These growing masses are then thought to divide with the help of “chaperones,” cellular proteins that aid in protein folding and transport, resulting in smaller prion particles called propagons. The propagons are then distributed to both mother and daughter cells during division, thereby infecting the next generation of cells. Though this theory of the prion life cycle was proposed a few years ago, scientists are still working out the underlying molecular mechanisms

As they report in this issue, Lev Osherovich and colleagues dissected yeast prions and found that growth and heritability are controlled by two independent and “portable” sequences. Furthermore, the heritability element seems to be the only thing that keeps slow growing protein aggregates from becoming infectious prions. Previous research showed that one end of the yeast protein, Sup35p, is critical for turning this normal housekeeping enzyme into a prion. The “prion-forming domain” of Sup35p consists of two segments: one stretch rich in the amino acids glutamine and asparagine and another made up of several, small series of amino acids, called oligopeptides. Osherovich and colleagues had earlier found another yeast protein, New1p, which had similar segments, though in reverse order.

To study the function of these sequences, the team constructed several strains of yeast, each with a small part of the prion-forming domain missing. By watching the behavior of these modified proteins, each fused to a green fluorescent protein for easy observation, the authors could infer the roles of the deleted segment.

For both Sup35p and New1p, the authors found that the area rich in glutamine and asparagine was responsible for the aggregation and growth of prions—acting like a patch of Velcro that locks the misshapen proteins together. While this had been suggested by previous research, the authors also found that this sticky sequence only adheres to proteins that mirror its own pattern of amino acids, thereby explaining why prions from different species don't often interact, a phenomenon called the species barrier. The stretch of oligopeptide repeats in Sup35p and New1p, however, was required for the inheritance of prions—the proper division of prion masses and subsequent distribution of propagons during cell division. The authors suggest that oligopeptide repeats function as a secure binding location for the chaperone proteins, which are necessary for heritability, and thus infectiousness, of prions. Their results also help to explain why stable inheritance of prions is rare; while many proteins have stretches of amino acids similar to the described aggregation sequence, few also contain sequences like oligopeptide repeats that permit inheritance.

Though both the aggregation sequence and the oligopeptide repeats are required for prion growth and infection, the segments seemed to function completely separately, allowing the authors to create a synthetic prion-forming domain by combining the aggregation element of New1p with the Sup35p replication/heritability element. This artificial prion acted like New1p, again showing that it is the sticky, aggregation element that specifies which proteins will be added to the growing prion mass. Osherovich and colleagues then went on to create another artificial prion by fusing the oligopeptide repeats to an expanded polyglutamine tract, the type of aggregation sequence responsible for the toxic buildup of brain proteins in Huntington's disease. With this simple addition, the slow growing aggregate was transformed into a heritable, infectious prion.

By creating artificial hybrid prions, Osherovich and colleagues showed that the two discrete elements of prion-forming domains are portable and work together regardless of their origins. The authors suggest that other artificial prions could be used as a model system to study different types of aggregation sequences, such as those found in the human prion protein responsible for Creutzfeldt-Jakob's disease or the misshapen plaques of proteins that contribute to Alzheimer's disease.

